# Prediction models for progression from diabetic kidney disease to end-stage renal disease: a systematic review and meta-analysis

**DOI:** 10.3389/fendo.2026.1812362

**Published:** 2026-05-20

**Authors:** Yang Shi, Zhaoxi Dong, Xiaomeng Shan, Xiqian Wang, Siyu Huang, Hongfang Liu, Linqi Zhang

**Affiliations:** 1 Treatment Center of Kidney Disease, The First Affiliated Hospital of Henan University of Chinese Medicine, Zhengzhou, China; 2 Dongzhimen Hospital, Beijing University of Chinese Medicine, Beijing, China; 3 Department of Endocrinology, The First Affiliated Hospital of Henan University of Chinese Medicine, Zhengzhou, China

**Keywords:** diabetic kidney disease, end-stage renal disease, meta-analysis, risk prediction model, systematic review

## Abstract

**Background:**

Diabetic kidney disease (DKD) is a major cause of end-stage renal disease (ESRD). Early identification of DKD patients at high risk of progressing to ESRD is essential, yet the overall performance, methodological quality, and translational readiness of prediction models for this transition remain unclear. To our knowledge, we conducted the first systematic review and meta-analysis focused specifically on prediction models for progression from established DKD to ESRD.

**Methods:**

We searched PubMed, Embase, Cochrane Library, Web of Science, China National Knowledge Infrastructure (CNKI), Wanfang, VIP Chinese Journal Service Platform, and the Chinese Biomedical Literature Database (CBM) for English- and Chinese-language studies published through 27 September 2025 that developed or validated DKD to ESRD prediction models. Two reviewers independently screened records and extracted data. Risk of bias was assessed using the updated PROBAST-AI checklist. Reported AUCs were pooled using random-effects meta-analysis (Stata 18.0) with 95% confidence intervals (CIs). We performed sensitivity analyses, assessed publication bias, and conducted subgroup analyses by inclusion of pathological predictors and prediction horizon.

**Results:**

Fifteen studies met inclusion criteria. PROBAST-AI judged all 15 studies at high risk of bias, mainly due to retrospective single-center designs, lack of blinded predictor assessment, use of predictors not routinely available in practice, and inadequate calibration and external validation; seven studies were limited to biopsy-proven DKD, limiting their applicability to routine clinical populations. Meta-analysis included training datasets from six studies (seven models) and validation datasets from three studies (three models). Pooled AUCs were 0.896 (95% CI, 0.853–0.940) for training models and 0.863 (95% CI, 0.803–0.923) for validation models. Five prespecified sensitivity analyses yielded broadly similar pooled AUCs, but interpretation remained exploratory because of persistent heterogeneity and universal high risk of bias. Subgroup analyses found no significant differences by pathological predictor inclusion or prediction horizon.

**Conclusions:**

Published DKD to ESRD models show promising discrimination in development and internal validation cohorts. However, pervasive methodological limitations, extreme heterogeneity, and scarce independent external validation severely restrict clinical generalizability. Prespecified sensitivity analyses yielded broadly similar pooled AUCs, but these results remain exploratory. Future prospective multicenter studies with rigorous external validation and calibration are urgently needed.

**Systematic Review Registration:**

https://www.crd.york.ac.uk/prospero/display_record.php?ID=CRD420251127778, identifier CRD420251127778.

## 1 Introduction

In 2021, an estimated 537 million people worldwide were living with diabetes mellitus, a figure projected to increase to 643 million by 2030 (1). Concomitant with this growing diabetes burden is a marked rise in diabetic kidney disease (DKD) (2), which has become the leading cause of end-stage renal disease (ESRD) (3). Once patients progress to ESRD, they require lifelong renal replacement therapy or transplantation, experience substantially reduced quality of life, and incur high health-care costs. The American Diabetes Association emphasizes that DKD management should prioritize prevention and early intervention, and that a core strategy is the early identification of patients at high risk of progression (4).

Risk prediction models integrate multiple risk factors with assigned weights to estimate an individual’s probability of developing a disease or experiencing an adverse outcome, thereby supporting clinical decision-making, resource allocation, and personalized interventions (5, 6). Traditional regression models perform well when predictor sets are modest in size and relationships are approximately linear, but they are less well suited to high-dimensional, nonlinear interactions. By contrast, machine-learning and other data-driven approaches can exploit complex, heterogeneous datasets and have therefore shown promise in DKD risk stratification (7, 8). At the same time, well-established tools developed for broad chronic kidney disease populations — for example, the Kidney Failure Risk Equation (KFRE) — have been validated across multiple centers and countries; however, their performance often requires recalibration when applied to DKD subpopulations, underscoring the rationale for DKD-specific predictive models (9).

Notably, prior systematic reviews and meta-analyses have summarized models that predict incident DKD among people with diabetes (10, 11). Yet, there remains a critical and neglected gap: no synthesis to date has systematically appraised and quantitatively pooled models that predict progression from established DKD to ESRD — a clinically pivotal transition. The primary studies published in recent years are growing in number but are highly heterogeneous with respect to study design, outcome definitions, prediction horizons, sample sources, and reporting quality. Moreover, reporting transparency and bias assessment are inconsistent, which prevents reproducible, fair comparisons and undermines confidence in model generalizability and translational readiness.

To our knowledge, we conducted the first systematic review and meta-analysis focused specifically on prediction models for progression from established DKD to ESRD. Our objective was to comprehensively evaluate model discrimination and calibration, to appraise methodological quality using contemporary tools, and to assess the models’ potential for clinical translation. By synthesizing existing evidence, we aim to identify robust predictors, expose recurrent methodological limitations, and provide guidance to improve future model development, external validation, and implementation.

## 2 Materials and methods

### 2.1 Study design

This systematic review and meta-analysis was conducted in accordance with the Preferred Reporting Items for Systematic Reviews and Meta-Analyses (PRISMA) statement (12). A completed PRISMA 2020 checklist is provided in **Supplementary Material**. The review objectives and data-extraction items were defined using the Checklist for Critical Appraisal and Data Extraction for Systematic Reviews of Prediction Modelling Studies (CHARMS) framework (13). The review protocol was prospectively registered in PROSPERO (CRD420251127778) on 15 September 2025 before data extraction began.

### 2.2 Data sources and search strategy

We performed comprehensive literature searches of PubMed, Embase, the Cochrane Library, Web of Science, China National Knowledge Infrastructure (CNKI), Wanfang Data, VIP Chinese Journal Service Platform, and the Chinese Biomedical Literature Database (CBM). The search was limited to studies published on or before 27 September 2025 and included publications in English and Chinese. The search strategy combined the use of MeSH terms and free words, supplemented by manual searches to include references traced back. The full search strategies are provided in the **Supplementary Material**.

### 2.3 Inclusion criteria

Eligibility was defined according to the PICOTS framework for prognostic model studies.

#### 2.3.1 Population

We included studies of adult patients diagnosed with DKD by clinical criteria or by renal biopsy pathology. No restrictions were placed on sex, age, ethnicity, geographic region, or diabetes type. Studies of non-DKD populations or studies that did not clearly distinguish DKD from other kidney diseases were excluded.

#### 2.3.2 Index prediction model

We included studies that developed or validated multivariable prediction models intended to estimate the risk of progression from DKD to ESRD. Eligible model types comprised traditional statistical models and machine-learning algorithms. Model formats included nomograms, risk scores, equations, or algorithmic implementations.

#### 2.3.3 Comparative models

No single comparator model was required. We included studies reporting one or more models and accepted within-study comparisons between models when presented.

#### 2.3.4 Outcome

The primary predicted outcome was progression to ESRD, defined as initiation of long-term renal replacement therapy, kidney transplantation, or persistent eGFR < 15 mL/min/1.73 m².

#### 2.3.5 Timing

No restriction was applied to the prediction horizon (time window) used by the models.

#### 2.3.6 Setting

Eligible studies were those in clinical care settings. Study designs included prospective or retrospective cohort studies, *post-hoc* analyses of randomized controlled trials, and case-cohort studies.

### 2.4 Exclusion criteria

We excluded reviews, meta-analyses, case reports, conference abstracts, clinical practice guidelines, narrative comments, expert opinions, letters, animal studies, and any study that did not present a multivariable prediction model.

### 2.5 Study selection

All search results were imported into EndNote for reference management and de-duplication. Two reviewers independently screened titles and abstracts for potential eligibility. Full texts of potentially relevant articles were retrieved and assessed against the inclusion and exclusion criteria. Disagreements were resolved by consensus or, when necessary, arbitration by a third independent reviewer.

### 2.6 Data extraction

Two reviewers independently extracted data using a standardized data extraction form. For studies that reported more than one model, data were extracted for each model separately. Extracted items included: first author, publication year, country, study type, DKD ascertainment method, sample size, median follow-up time, validation method, modelling method, calibration methods, model performance metrics, and the final predictors included in the model. The area under the receiver operating characteristic curve (AUC) and its 95% confidence interval (CI) were extracted as the primary performance metric. When necessary, study authors were contacted to obtain missing or clarifying information.

### 2.7 Risk of bias and applicability assessment

We assessed methodological quality using the updated Prediction model Risk Of Bias Assessment Tool for Artificial Intelligence (PROBAST-AI) checklist, which covers four domains: participants, predictors, outcomes, and analysis (14). Each signaling question was rated as “yes”, “probably yes”, “no”, “probably no”, or “no information”. A domain was judged at high risk of bias if any key signaling item was rated “no” or “probably no”; overall risk of bias was rated low only if all domains were judged low risk. Applicability was assessed across the participants, predictors, and outcome domains using the same approach. Prior to formal assessment, two evidence-trained reviewers discussed PROBAST-AI items to harmonize interpretation; five randomly selected studies were jointly rated to ensure inter-rater consistency. Two reviewers then independently rated all included studies; disagreements were resolved by consensus.

### 2.8 Statistical analysis

Meta-analyses were performed using STATA 18.0. Given the anticipated high heterogeneity and high risk of bias, the quantitative synthesis was prespecified as exploratory rather than confirmatory. Between-study heterogeneity was quantified with the I² statistic, with thresholds of 25%, 50%, and 75% corresponding to low, moderate, and high heterogeneity, respectively. We selected fixed-effect or random-effects models according to heterogeneity: random-effects models were used when I² > 50%; otherwise, fixed-effect models were used (15). For the primary analysis, when studies reported multiple candidate models, we selected the author-designated final model; if no final model was specified, we selected the model with the longest clinically relevant prediction horizon (16). We pooled AUCs and their 95% CIs separately for model training datasets and for validation datasets; pooled estimates were displayed in forest plots (17). For interpretative purposes, AUC values were categorized as follows: < 0.60, fail; 0.60–0.70, poor; 0.70–0.80, fair; > 0.80, considerable; > 0.90, excellent (18). We assessed the influence of individual studies on pooled estimates using a leave-one-out sensitivity analysis. Publication bias was examined with Egger’s linear regression test (19). To explore sources of heterogeneity, we performed subgroup analyses stratified by (1) inclusion of renal pathological predictors and (2) prediction horizon, using prespecified time windows of 1, 2, 3, and 5 years. Subgroup pooling used the same model-selection rules described above (fixed vs random effects determined by I²). Between-group differences were tested by meta-regression within a random-effects framework. To test the robustness of the pooled estimates, we conducted five sensitivity analyses (1): excluding biopsy-proven DKD cohorts (2), restricting the analysis to the composite-endpoint subgroup (3), restricting the analysis to models based on routinely available clinical predictors while excluding renal pathology predictors (4), applying an alternative within-study model-selection rule that prioritized the shortest clinically relevant prediction horizon and, when multiple models shared the same horizon, the first model reported, and (5) excluding the study with the most concerning overall risk-of-bias profile among the pooled studies. Statistical significance was set at p < 0.05.

## 3 Results

### 3.1 Study selection

**Figure 1:**
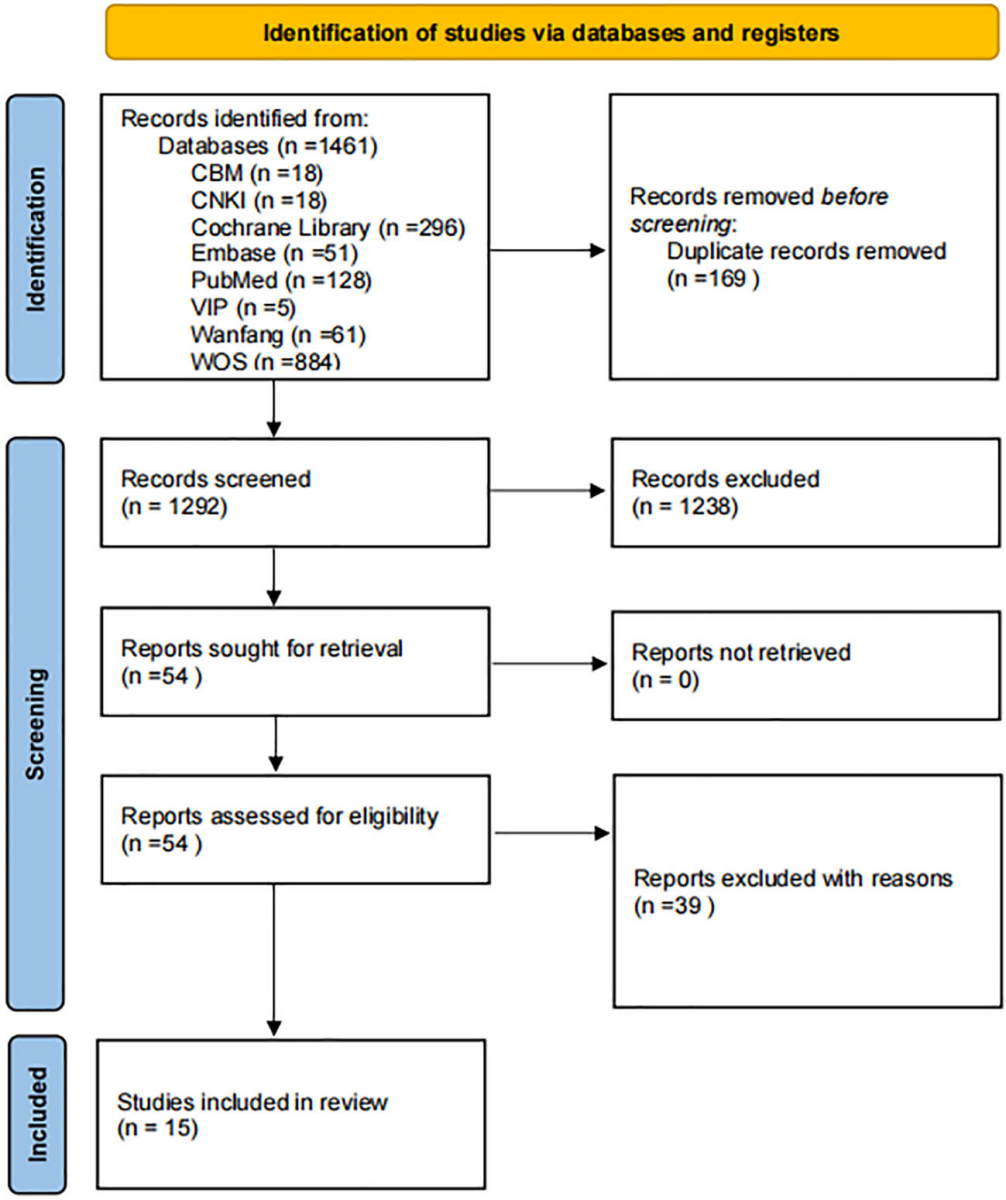
Study search and selection flowchart. Flowchart according to the preferred reporting items for systematic reviews and meta-analyses (PRISMA 2020). n= number of studies/records/reports.

The database search yielded 1,461 records. After duplicate removal in EndNote, 1,293 records remained. Title and abstract screening reduced the pool to 54 full-text articles for assessment. After full-text review, 15 studies met the inclusion criteria and were included in the review (8, 20–33). Among the 39 full-text articles excluded after detailed assessment, Zhao et al. (2022) was excluded because it primarily evaluated the incremental prognostic value of glomerular lesion severity added to anemia status and clinical data in a retrospective biopsy-confirmed diabetic nephropathy cohort, rather than developing or validating a standalone DKD-to-ESRD multivariable prediction model suitable for quantitative synthesis (34). The remaining exclusions were mainly due to non-DKD populations, non-ESRD outcomes, or the absence of a multivariable prediction model. The PRISMA flow diagram is shown in **Figure 1**.

### 3.2 Study characteristics

Fifteen studies were included. The earliest study was published in 2006; ten studies were published within the most recent five years. Eleven studies were conducted in China, two in Japan, one in the United Kingdom, and one was a multinational study. Fourteen studies had retrospective cohort designs, and one study was a *post-hoc* analysis of a prospective randomized controlled trial. Although all studies used progression to ESRD as the primary endpoint, the operational definitions varied across studies and were classified into three principal types (1): treatment-based definitions (e.g., initiation of long-term renal replacement therapy for >3 months or receipt of kidney transplantation); (2) eGFR-threshold definitions (e.g., persistent eGFR < 15 mL/min/1.73 m²); and (3) composite endpoints combining eGFR thresholds, initiation of renal replacement therapy, or kidney-related death. Detailed study characteristics are presented in **Table 1**.

**Table 1:** Basic characteristics of the included literature.

Author	Year of publication	Country of the author	Study type	Diagnostic method	Prediction outcome	Reported adherence to TRIPOD/TRIPOD-AI
Yu et al, 2025 (20)	2025	China	Single-center retrospective cohort study	Biopsy-proven	Requirement of permanent renal replacement therapies for >3 months	No
Zou et al.2022 (8)	2022	China	Single-center retrospective cohort study	Biopsy-proven	eGFR <15 mL/min/1.73 m² or requirement of renal replacement therapy	No
Sun et al., 2020 (21)	2020	China	Single-center retrospective cohort study	Biopsy-proven	Death due to diabetes with renal manifestations or renal failure;Hospitalization due to nonfatal renal failure;eGFR < 15 ml/min/1.73 m²	Yes
Cheng, 2020 (22)	2020	China	Single-center retrospective cohort study	clinically diagnosed	Renal replacement therapy (including hemodialysis, peritoneal dialysis, and kidney transplantation)	No
Wang, 2022 (23)	2022	China	Single-center retrospective cohort study	Biopsy-proven	All-cause death, initiation of dialysis, or renal transplantation	No
He, 2021 (24)	2021	China	Single-center retrospective cohort study	clinically diagnosed	eGFR < 15 ml/min/1.73m²	No
Zhou, 2023 (25)	2023	China	Single-center retrospective cohort study	clinically diagnosed	eGFR < 15 ml/min/1.73m²	No
Xue, 2023 (26)	2023	China	Single-center retrospective cohort study	clinically diagnosed	eGFR < 15 ml/min/1.73m² or initiation of renal replacement therapy	No
Lin et al., 2025 (27)	2025	China	Multi-center retrospective cohort study	Biopsy-proven	eGFR <15 mL/min/1.73m², initiation of long-term dialysis, or kidney transplantation	No
Hu et al.2025 (28)	2025	China	Single-center retrospective cohort study	Cohort 1: clinically diagnosed Cohort 2: biopsy-proven	eGFR < 15 mL/min/1.73 m² and/or initiation of renal replacement therapy.	No
Goubar et al, 2024 (29)	2024	United Kingdom	Multi-center retrospective cohort study	clinically diagnosed	eGFR <15 mL/min/1.73m²	No
Hoshino et al, 2015 (30)	2015	Japan	Single-center retrospective cohort study	Biopsy-proven	Initiation of chronic dialysis	No
Tu et al, 2021 (31)	2021	China	Single-center retrospective cohort study	clinically diagnosed	entering the ESRD period	Yes
Keane et al, 2006 (32)	2006	Multinational	*Post-hoc* analysis of a randomized controlled trial	clinically diagnosed	Need for long-term dialysis or renal transplantation	No
Yamanouchi et al, 2018 (33)	2018	Japan	Multi-center retrospective cohort study	Biopsy-proven	Initiation of hemodialysis or peritoneal dialysis, or renal transplantation, or death from uremia	No

### 3.3 Model development approaches and performance

Sample sizes of included studies ranged from 140 to 7,296 participants. Across included cohorts, the reported incidence of progression from DKD to ESRD varied markedly, ranging from 10.10% to 65.50%. All studies used conventional regression methods (logistic regression or Cox proportional hazards regression) for model development; two studies (8, 23) additionally evaluated multiple machine-learning algorithms (e.g., random forest, support vector machine, XGBoost) for comparative purposes. All 15 studies reported the final model format, which included nomograms, risk score equations, machine-learning models, and risk assessment tables. Reported discrimination (AUC or C-index) ranged from 0.742 to 0.98. Regarding validation, nine studies reported internal validation strategies (e.g., cross-validation or training/validation splits), and one study (29) performed an independent external validation of an existing model (KFRE). Further details on modelling approaches and performance metrics are summarized in **Table 2**.

**Table 2:** Model construction method and predictive performance.

Study	Modeling dataset	Validation dataset	Median follow-up time	ESRD incidence rate	Variable selection	Validation method	Modeling methods	Internal validation methods	Model presentation format	AUC		Calibration
										Training	Validation	
Yu et al, 2025 (20)	155	46	31 months	27.10%	Combination of VIMP and minimum depth method	A	C	S	Nomogram	①:0947 ②:0867 ③:0905	①:0.888 ②:0939 ③:0886	/​
Zou et al, 2022 (8)	390	98	3 years	40.51%	Machine learning algorithms for feature selection	A	D、E、F、G	V	Machine learning model、Nomogram	/​	①:0.90 ②:0.88 ③:0.88 ④:0.83 ⑤:0.84	/​
Sun et al, 2020 (21)	478	119	/	47.10%	Univariate logistic regression, followed by multivariable logistic regression with backward selection	A	D	S	Nomogram	①:0.864 ②:0.865(0.863–0.867) ③:0.842 ④:0.866	①:0.870 ②:0.876 (0.874–0.878) ③:0.849 ④:0.875	W
Cheng, 2020 (22)	641	641	3 years	42.4%​	Univariate logistic regression followed by backward stepwise multivariate logistic regression	A	D	U	Nomogram	①:0.57 ②:0.76 ③:0.92 ④:0.96 ⑤:0.98 ⑥:0.98 ⑦:0.98 ⑧:0.98	①:0.52 ②:0.76 ③:0.91 ④:0.95 ⑤:0.98 ⑥:0.98 ⑦:0.98 ⑧:0.96	W
Wang,2022 (23)	203	41	4.4 years	40.50%	Univariate and multivariate Cox regression analysis; Supervised machine learning algorithms with feature importance analysis.	A	D、E、H、F、I	U	Machine learning model、Model equation	/​	①:0.840 ②:0.763 ③:0.808 ④:0.6975 ⑤:0.870 ⑥:0.870 ⑦:0.773 ⑧:0.841	/​
He, 2021 (24)	5,452	1,658​	/​	10.10%	Univariate logistic regression and multivariate logistic backward stepwise regression	A	D	S	Model equation	0.942(95% CI: 0.934–0.949)	0.912(95% CI: 0.897–0.925)	W
Zhou, 2023 (25)	801	50	/​	/	Univariate and multivariate logistic regression analysis	A	D	S	Model equation	0.888	0.936	/​
Xue, 2023 (26)	519	519	32 months	29.9%​	Univariate and multivariate COX regression analysis	A	C	/​	Nomograms	①:0.909 (0.867-0.951) ②:0.881 (0.841-0.920) ③:0.881 (0.843-0.919)	/​	/​
Lin et al, 2025 (27)	140	140	12 months	57.9%​	Univariate Cox regression followed by multivariate Cox regression (stepwise forward LR)	A	C	/​	Nomograms	①:0.898(0.839-0.958) ②:0.889(0.818-0.959) ③:0.876(0.785-0.968) ④:0.893(0.796-0.990)	/​	X
Hu et al, 2025 (28)	patients with clinically diagnosed DKD: 555 patients with biopsy-proven DKD: 86	patients with clinically diagnosed DKD: 555 patients with biopsy-proven DKD: 86	38.0 months 32.0 months	patients with clinically diagnosed DKD:31.7% patients with biopsy-proven DKD:35.3%	Univariate Cox regression followed by multivariate Cox regression	A	C	/​	Model equation	patients with clinically diagnosed DKD: ①:0.901(0.851-0.938)②:0.894(0.859-0.928)③:0.887(0.852-0.921) ④:0.897(0.847-0.939)⑤:0.890(0.853-0.922)⑥:0.882(0.843-0.917) patients with biopsy-proven DKD: ①:0.851(0.672-0.929)②:0.611(0.350-0.864) ③:0.822(0.668-0.943)④:0.710(0.418-0.971) ⑤:0.854(0.668-0.974)⑥:0.731(0.483-0.947) ⑦:0.809(0.612-0.961)⑧:0.751(0.495-0.965)	/​	/​
Goubar et al, 2024 (29)	Full cohort:7,296 complete case analysis dataset:5,930	Full cohort:7,296 complete case analysis dataset:5,930	10.2 years	Full cohort:15.5% complete case analysis dataset:/	/	B	C	/	Model equation	/​	Full cohort:①:0.843 (0.839–0.847)②:0.801 (0.798–0.804) complete case analysis dataset:①:0.844 (0.815–0.874)②:0.799 (0.776–0.822)	X
Hoshino et al, 2015 (30)	205​	205​	5.57 years	/	Multivariate Cox regression with bootstrap inclusion fractions	A	C	V	Risk assessment form + Model equation	①:0.7802 (0.7185-0.8419) ②: 0.8162 (0.7588–0.8736) ③:0.8637 (0.8155–0.9120) ④:0.8687 (0.8199–0.9175) ⑤:0.9013 (0.8608–0.9418) ⑥:0.9091 (0.8710–0.9473) ⑦:0.9317 (0.8984–0.9649)	/​	/​
Tu et al, 2021 (31)	438	438	63.1 months​	45.90%	Univariate and multivariate Cox regression analysis	A	C	/​	Nomograms	0.742	/​	/​
Keane et al, 2006 (32)	1,513​	1,513​	3.4 years	22.50%	Backward selection (P < 0.01) from multivariate Cox regression	A	C	T	Model equation + Risk assessment form	/​	/​	/​
Yamanouchi et al, 2018 (33)	296	98	1.9 years	65.50%	Use of pre-existing scores/variables	A	C	S	Model equation、Risk assessment form	①:0.776 ②:0.672 ③:0.790	①:0.747 ②:0.666 ③:0.753	/​

A, internal validation set; B, external validation set; C, Cox regression; D, logistic regression; E, support vector machine (SVM); F, random forest; G, gradient boosting machine (GBM); H, XGBoost; I, AdaBoost; S, random split; T, jackknife method; U, five-fold cross-validation; V, ten-fold cross-validation; W, Hosmer–Lemeshow test; X, calibration curve;/= not mentioned.

### 3.4 Predictors included in the models

**Figure 2:**
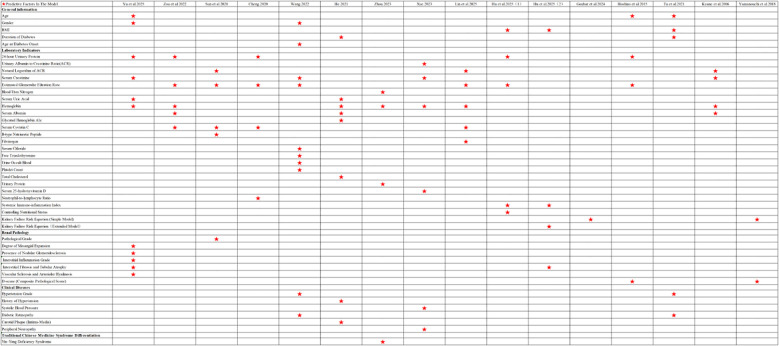
Distribution of predictor variables in the model. ✩: Predictive factors in the model.

All 15 studies reported the final model format; formats included nomograms, risk-score equations, machine-learning models, and tabulated risk tools. Variable selection strategies varied (**Table 2**). The most common approach was conventional statistical selection, typically univariable screening followed by multivariable stepwise regression (forward or backward selection). Two studies (8, 23) applied machine-learning methods for feature selection. One study (33) adopted variables from an existing score (KFRE) directly. Predictors were grouped into five categories: general information, laboratory indicators, renal pathology, clinical diseases, and Traditional Chinese Medicine syndrome differentiation. The most frequently included predictors were estimated glomerular filtration rate (eGFR), hemoglobin, 24-hour urinary protein, serum cystatin C, and serum creatinine (Scr). The distribution of predictors across studies is illustrated in **Figure 2**.

### 3.5 Risk of bias and applicability assessment

**Figure 3:**
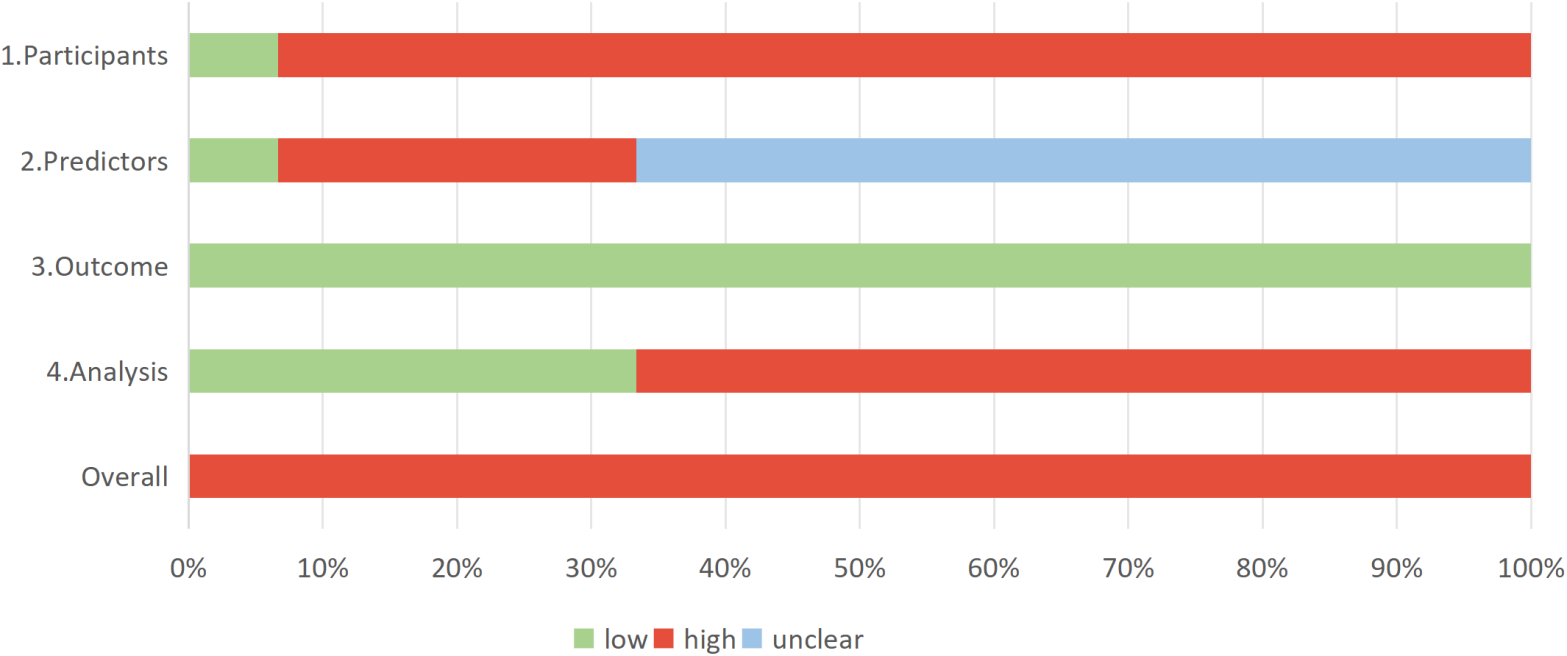
Risk of bias graph.

The PROBAST-AI assessments are reported in **Table 3**. Overall, all included studies were judged to be at high risk of bias (**Figure 3**). Common sources of high risk included retrospective design (leading to selection bias), single-center recruitment with limited sample representativeness, lack of model calibration, and insufficient external validation. For predictor assessment, the majority of studies did not state whether predictor measurement was performed blinded to outcome, raising the possibility of information bias. Four studies incorporated renal pathology variables in their final models; because these pathological measures are not routinely available in typical clinical screening or management settings, their inclusion contributed to elevated risk-of-bias judgments. In the analytical domain, ten studies did not report model calibration; six studies used cross-validation or a separate external validation dataset, while five relied solely on simple random splits of available data. Many studies failed to specify how continuous predictors were modeled, and performance reporting was frequently limited to discrimination measures without comprehensive calibration or clinical utility assessment.

**Table 3:** Assessment of bias and applicability in the included model.

Study	ROB				Applicability			Overall	
	Participants	Predictors	Outcome	Analysis	Participants	Predictors	Outcome	ROB	Applicability
Yu et al, 2025 (20)	①	①	②	①	⑤	⑤	④	①	⑤
Zou et al, 2022 (8)	①	③	②	①	⑤	④	④	①	⑤
Sun et al.2020 (21)	①	①	②	②	⑤	⑤	④	①	⑤
Cheng.2020 (22)	①	③	②	②	④	④	④	①	④
Wang.2022 (23)	①	③	②	①	⑤	④	④	①	⑤
He.2021 (24)	①	③	②	②	④	④	④	①	④
Zhou.2023 (25)	①	③	②	①	④	④	④	①	④
Xue.2023 (26)	①	③	②	①	④	④	④	①	④
Lin et al.2025 (27)	①	③	②	①	⑤	④	④	①	⑤
Hu et al.2025 (28)	①	③	②	①	④	④	④	①	④
Goubar et al.2024 (29)	①	③	②	②	④	④	④	①	④
Hoshino et al.2015 (30)	①	①	②	①	⑤	⑤	④	①	⑤
Tu et al.2021 (31)	①	③	②	①	④	④	④	①	④
Keane et al.2006 (32)	②	②	②	①	④	④	④	①	④
Yamanouchi et al.2018 (33)	①	①	②	②	⑤	⑤	④	①	⑤

①, high risk of bias; ②, low risk of bias; ③= unclear risk of bias; ④= high applicability; ⑤= low applicability.

Seven studies (20, 21, 23, 27, 28, 30, 33) were judged to have low applicability because their study populations were restricted to biopsy-proven DKD patients. Since patients undergoing renal biopsy are a selected subgroup in clinical practice, models developed solely in biopsy cohorts may not generalize to broader, clinically diagnosed DKD populations.

### 3.6 Meta-analysis results

The primary analysis retained one model per study according to the prespecified selection rule described above. One study reported separate cohorts defined by clinical diagnosis versus pathological diagnosis; owing to substantive differences in patient characteristics and predictors, we extracted performance measures for both cohorts separately. After excluding 8 studies that did not report AUCs or 95% CIs, 6 studies (7 models) based on training datasets and 3 studies (3 models) based on validation datasets were included in the meta-analysis.

#### 3.6.1 Training-set models

**Figure 4:**
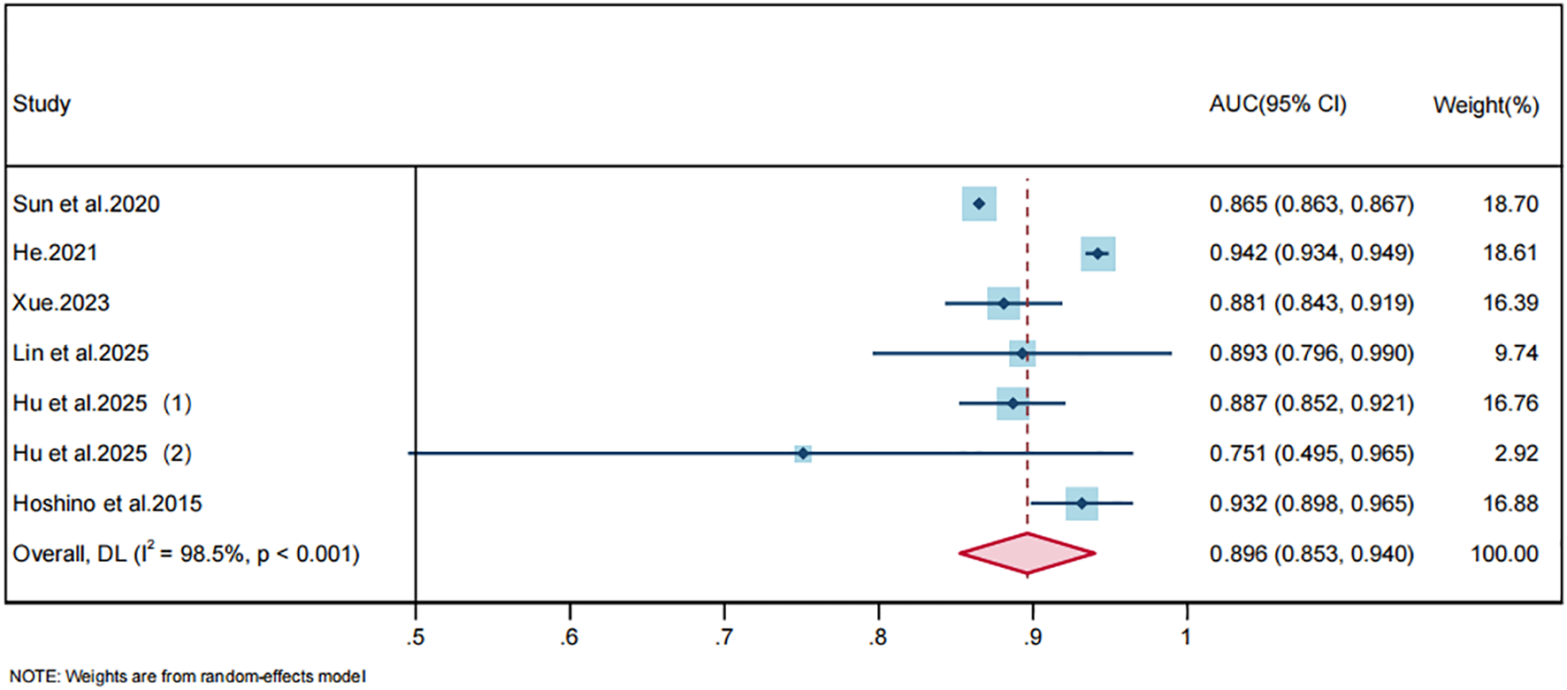
Forest plot of the random effects meta-analysis of pooled AUC estimates for 7 training-set models. Note: Parentheses following one study denote distinct models derived from that study.

Heterogeneity among the training-set AUCs was very high (I², 98.5%, P < 0.001); therefore, we used a random-effects meta-analysis. The pooled AUC for training sets was 0.896 (95% CI, 0.853–0.940), indicating excellent discrimination. The corresponding forest plot is shown in **Figure 4**. Leave-one-out sensitivity analyses demonstrated robustness: pooled AUC estimates varied between 0.887 and 0.909 after omission of any single study, and all resulting 95% CIs substantially overlapped the primary pooled estimate. Egger’s regression test yielded an intercept of 3.589 (95% CI, −5.726 to 12.904; t, 0.99; P, 0.367), providing no evidence of small-study effects or publication bias.

#### 3.6.2 Validation-set models

**Figure 5:**
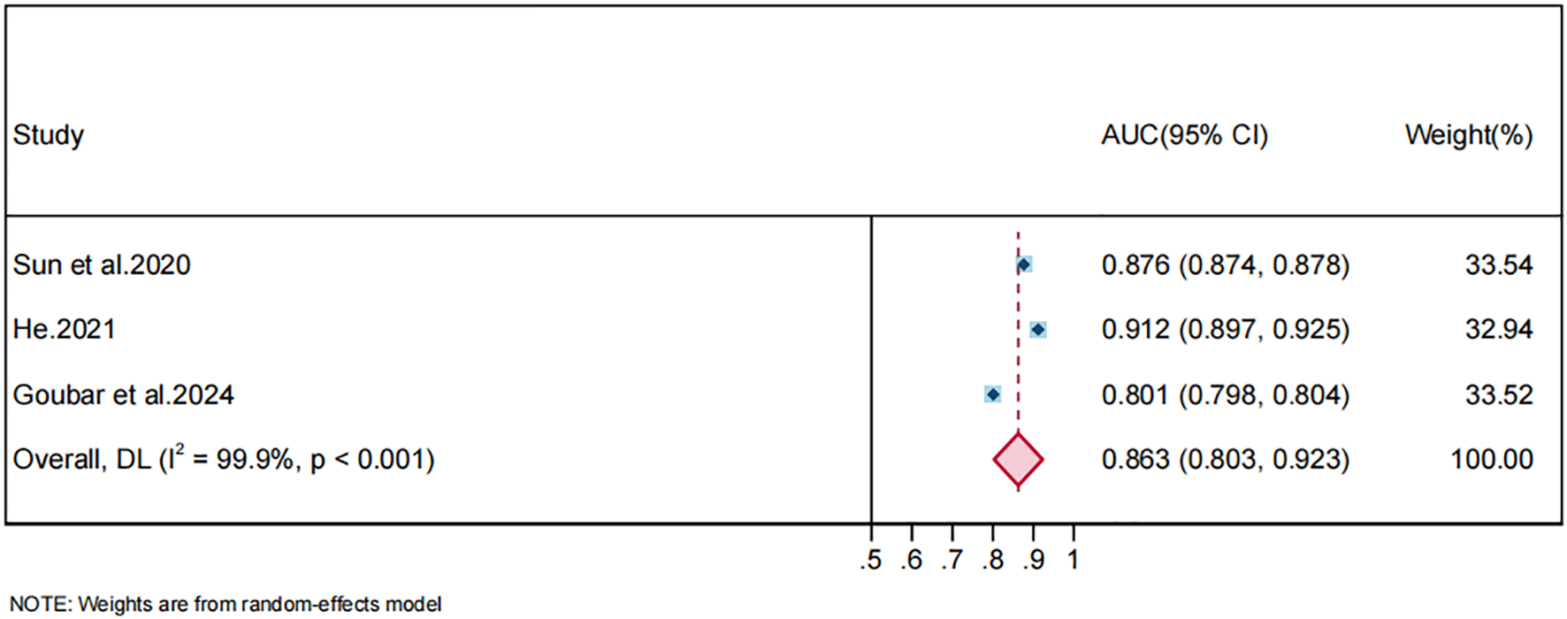
Forest plot of the random effects meta-analysis of pooled AUC estimates for 3 validation-set models.

Heterogeneity among validation-set AUCs was also very high (I², 99.9%, P < 0.001); a random-effects model was therefore applied. The pooled AUC for validation sets was 0.863 (95% CI, 0.803–0.923). This estimate was close to the pooled training−set AUC, suggesting that the models retained similar discriminative performance in independent data; however, the small number of validation studies and their high risk of bias limit the strength of this conclusion. The corresponding forest plot is shown in **Figure 5**. Given the small number of validation studies, further sensitivity analyses, subgroup analyses or publication bias assessments were not performed for validation-set data.

#### 3.6.3 Subgroup analyses

Two subgroup analyses were performed on the training-set data to explore potential sources of heterogeneity. When stratified by the inclusion of renal pathology predictors, models incorporating pathology had a pooled AUC of 0.887 (95% CI, 0.825–0.950; I², 87.7%), whereas models including only clinical predictors had a pooled AUC of 0.898 (95% CI, 0.860–0.936; I², 82.1%). Meta-regression indicated no statistically significant difference between these subgroups (β, 0.0106, P, 0.770).

We stratified models by prespecified prediction windows of 1, 2, 3, and 5 years. The pooled AUCs were as follows: 1-year models, 0.904 (95% CI, 0.877–0.931); 2-year models, 0.880 (95% CI, 0.846–0.914); 3-year models, 0.887 (95% CI: 0.863–0.912); and 5-year models, 0.885 (95% CI, 0.853–0.917). Heterogeneity within each subgroup was negligible (I², 0% for all). Using the 1-year group as the reference, meta-regression (overall F-test) yielded P, 0.702, indicating no statistically significant differences in discriminative performance across the examined prediction horizons.

#### 3.6.4 Sensitivity analyses

To assess robustness, we conducted five prespecified sensitivity analyses (**Table 4**). Excluding biopsy-proven DKD cohorts, that is, restricting the analysis to clinically diagnosed DKD, yielded a pooled AUC of 0.906 (95% CI, 0.860–0.952; I², 89.0%). Restricting the analysis to models based on routinely available clinical predictors, that is, excluding renal pathology predictors, produced a pooled AUC of 0.898 (95% CI, 0.860–0.936; I², 82.1%), essentially identical to the primary analysis. Under the alternative within-study model-selection rule, the pooled AUC was 0.883 (95% CI, 0.840–0.927; I², 98.5%), slightly lower than the primary estimate. Excluding the study with the most concerning overall risk-of-bias profile among the pooled studies, Hoshino et al., 2015 (30), yielded a pooled AUC of 0.889 (95% CI, 0.841–0.937; I², 98.7%), which was very similar to the primary estimate. When stratified by ESRD definition, the dialysis-based subgroup (n, 1) yielded an AUC of 0.9317 (95% CI, 0.8984–0.9649), and the eGFR-threshold subgroup (n, 1) yielded an AUC of 0.942 (95% CI, 0.934–0.949); because each subgroup contained only one study, these results were summarized descriptively rather than pooled. The composite-endpoint subgroup (n, 5) yielded a pooled AUC of 0.865 (95% CI, 0.863–0.867; I², 0.0%), although this estimate was largely driven by a single study with an extremely narrow confidence interval. Collectively, these analyses suggest that the main findings were broadly stable, although the pooled estimates should be interpreted cautiously in view of persistent heterogeneity and high risk of bias.

**Table 4:** Summary of primary and sensitivity analyses of pooled AUCs for training-set models.

Analysis	No. of models	Pooled AUC (95% CI)	I² (%)	Compared with primary analysis
Primary analysis	7	0.896 (0.853–0.940)	98.5	Reference
Excluding biopsy-proven DKD cohorts	3	0.906 (0.860–0.952)	89.0	Slightly higher
ESRD definition: composite endpoint only	5	0.865 (0.863–0.867)	0.0	Lower, but dominated by one study
Clinical-predictor-only models	6	0.898 (0.860–0.936)	82.1	Essentially unchanged
Alternative model-selection rule	7	0.883 (0.840–0.927)	98.5	Slightly lower
Excluding the study with the most concerning risk-of-bias profile	6	0.889 (0.841–0.937)	98.7	Essentially unchanged

AUC, area under the receiver operating characteristic curve; CI, confidence interval; I², percentage of total variation attributable to between-study heterogeneity. Primary analysis used the author-designated final model; when no final model was specified, the model with the longest clinically relevant prediction horizon was selected. Excluding biopsy-proven DKD cohorts, restricting to clinically diagnosed DKD. ESRD definition: composite endpoint only, pooled analysis limited to studies defining ESRD as a composite endpoint; the dialysis-based and eGFR-threshold–based subgroups each contained one study and were therefore summarized descriptively in the text rather than pooled quantitatively. Clinical-predictor-only models excluded renal pathology predictors. Alternative model-selection rule prioritized the shortest clinically relevant prediction horizon and, when multiple models shared the same horizon, the first model reported. Excluding the study with the most concerning risk-of-bias profile, removing Hoshino et al., 2015 (30) from the primary training-set meta-analysis.

## 4 Discussion

This systematic review and meta-analysis comprehensively evaluated published multivariable models that predict progression from diagnosed DKD to ESRD. The pooled discrimination was promising, but because these estimates were derived from studies at high risk of bias, they should be interpreted as exploratory. In addition, the literature exhibited marked methodological heterogeneity and an overall paucity of rigorous external validation, both of which substantially limit the models’ clinical generalizability and translational potential.

### 4.1 Principal predictors: from canonical renal indices to multidimensional integration

Across the 15 included studies, predictor sets evolved from reliance on classical renal function indices toward multidimensional combinations of clinical, pathological, and biomarker information. First and foremost, measures of kidney function and proteinuria remain foundational (35). eGFR or Scr were key predictors in 10 studies (8, 20–23, 26–28, 30, 32), and proteinuria metrics (e.g., 24-hour urinary protein, urinary albumin to creatinine ratio) were likewise used in 10 studies (8, 20–22, 25–28, 30, 32). The KFRE, which integrates eGFR and urinary albumin to creatinine ratio, was validated or used as a comparator in three studies included here (28, 29, 33), underscoring the central role of these two variable classes.

Serum cystatin C appeared in four studies (8, 21, 22, 27) and may confer incremental predictive value because it can detect declines in filtration earlier and more reliably than creatinine-based measures in some cohorts (36, 37). Markers of anemia and nutritional status were also recurrent: hemoglobin featured in seven studies (8, 20, 24–27, 32), serum albumin in three studies (8, 24, 32), and one study reported a CONUT score that includes albumin (28). These variables likely reflect systemic reserve and catabolic state that are relevant to disease progression (38–41).

Inflammation-related indices emerged as additional signals: the neutrophil-to-lymphocyte ratio appeared in one included study (21), and the systemic immune-inflammation index in another (28), suggesting that chronic systemic inflammation contributes to DKD progression and provides measurable prognostic information (42, 43). Metabolic and cardiovascular factors — notably hyperuricemia (20, 24), dyslipidemia (24), hypertension (23, 24, 26, 31), and diabetes duration (24, 31) — were also commonly selected, consistent with established epidemiologic links to renal decline (44–48).

Several investigations incorporated renal biopsy pathology (pathologic grading or composite D-scores), aiming to improve precision; five studies did so (20, 21, 28, 30, 33). Although pathology-enhanced models often outperformed clinical-only models within the same cohort, our subgroup meta-analysis found no statistically significant pooled advantage for pathology-inclusive models (pooled AUC, 0.887) versus clinical-only models (pooled AUC, 0.898; P, 0.770). This discrepancy likely reflects heterogeneity in pathology scoring systems, selection bias of biopsy cohorts, and methodological differences in model construction. Accordingly, the standardized application of pathological metrics and optimized strategies for integrating histopathology with clinical and biomarker data remain unresolved issues requiring further research — for example, via harmonized scoring, centralized reading, or quantitative digital pathology pipelines (49).

### 4.2 Model performance, validation, and translational barriers

Overall discrimination across the included models was generally good. Twelve studies (8, 20–30) reported AUC or C-index values greater than 0.8, and four studies (22, 24, 25, 30) reported AUCs exceeding 0.9. Meta-analysis yielded a pooled AUC of 0.896 (95% CI, 0.853–0.940) for training datasets and 0.863 (95% CI, 0.803–0.923) for validation datasets, indicating generally promising discriminative performance in development and internal validation cohorts, although these estimates should be interpreted with caution given the high risk of bias and extreme heterogeneity. Subgroup analysis by prediction horizon showed that model discrimination remained broadly stable across short-to-mid term windows (1–5 years), with no evidence of performance decay as the prediction window increased. However, discrimination alone is insufficient to establish clinical usefulness: the AUC reflects ranking ability but does not assess calibration or clinical net benefit (50, 51).

The sensitivity analyses indicated that the pooled AUC was broadly stable after excluding biopsy-only cohorts, restricting the analysis to clinically available predictors, and excluding the study with the most concerning risk-of-bias profile. The alternative model-selection rule yielded a slightly lower pooled AUC. Together with the extreme between-study heterogeneity and uniformly high risk of bias, these findings indicate that pooled AUCs should be interpreted as exploratory summaries rather than definitive estimates of clinical performance.

Similar prediction-model reviews have also pooled AUCs under conditions of high risk of bias and substantial heterogeneity, while interpreting pooled estimates cautiously as descriptive summaries rather than definitive performance measures. For example, Xu et al. reported that all included DKD prediction models were at high risk of bias (10), and Luo et al. found that most epilepsy prediction studies were at high risk of bias, with heterogeneity remaining substantial across datasets (52). In prediction-model meta-analysis, between-study heterogeneity is expected because performance varies across populations, settings, and prediction horizons, and random-effects meta-analysis is commonly used to summarize average performance while quantifying that heterogeneity. These examples support the use of exploratory quantitative synthesis, provided that its limitations are stated explicitly.

External validation was uncommon, and recalibration was rarely performed. Only one study (29) conducted an independent external validation of an existing model (KFRE) in a multiethnic cohort; the remainder were primarily development studies. While many development studies carried out internal validation (cross-validation or train/test splits), the near-absence of systematic external testing constrains claims of generalizability across diverse populations and healthcare settings. Only five studies (21, 22, 24, 27, 29) reported calibration assessments (calibration plots or Hosmer–Lemeshow tests). Evidence from prior multicenter validations indicates that models can exhibit calibration drift across regions and etiologies, so local recalibration or model updating is usually necessary before deployment in a new setting (53).

The very high heterogeneity observed in this study may be partly attributable to inconsistent definitions of ESRD across the included studies (54). Definitions ranged from purely clinical hard endpoints (for example, initiation of dialysis) to laboratory thresholds based on eGFR, and to composite endpoints that included death; these approaches differ in event incidence, ascertainment criteria, and the challenges they pose to prediction models. For example, eGFR-based thresholds may capture endpoint events earlier and more frequently, whereas treatment-initiation definitions more closely reflect clinical decision points but can be affected by local healthcare availability. Such definitional inconsistency imposes inherent limitations on directly comparing or pooling AUCs across studies (55).

Methodological quality varied and introduced multiple sources of bias. All 15 studies were retrospective, which raises unavoidable selection and information-bias risks. Eleven studies were single-center (8, 20–26, 28, 30, 31), limiting representativeness. Importantly, eight studies (8, 20, 21, 23, 27, 28, 30, 33) defined DKD by renal biopsy. Patients undergoing biopsy represent a selected subgroup who more often have atypical presentations, more severe disease, or rapid progression; consequently, models derived from biopsy cohorts may not generalize to the broader population of clinically diagnosed DKD patients (56–58). One study explicitly modeled clinical-diagnosis and pathology-confirmed cohorts separately, which is a methodologically useful approach to this problem.

Our methodological appraisal further revealed limitations at the level of predictor ascertainment. First, most studies did not explicitly state whether predictor data were collected or extracted blind to outcome; in retrospective designs, awareness of patient outcomes by assessors can introduce unconscious bias during data extraction or interpretation, undermining the objectivity of observed predictor–outcome associations. Second, several models included predictors that are not routinely obtainable in the intended clinical setting—most notably renal pathology variables. Although these measures may enhance performance within specific cohorts, their dependence on invasive kidney biopsy substantially restricts the practicality and immediate availability of such models for widespread DKD screening and risk stratification (59, 60).

Sample sizes and follow-up varied substantially across studies. Sample sizes ranged from 140 to 7,296, and most studies did not report formal sample-size calculations. Contemporary methodological guidance recommends that sample-size planning for prediction-model development should move beyond simple events-per-variable (EPV) heuristics and account for expected event rate, anticipated model performance, the number of candidate predictors, and model complexity to reduce overfitting risk and ensure adequate information for calibration assessment (61, 62). Furthermore, four studies (25, 26, 31, 32) did not report median follow-up time, and four studies (20, 23, 27, 33) had median follow-up shorter than 3 years. Short follow-up can fail to capture long-term progression to ESRD and may reduce a model’s ability to predict distant outcomes.

Regarding modelling techniques, 12 studies (20–22, 24–28, 30–33) used conventional regression methods (Cox or logistic regression), while two studies (8, 23) applied machine-learning algorithms and reported potential performance gains. Variable selection strategies varied and included traditional univariable screening followed by stepwise multivariable regression, as well as ML-based feature-importance ranking. Missing data strategies varied from complete-case deletion (25) to multiple imputation (8, 21, 22, 29), with multiple imputation being the preferred approach. Adherence to reporting guidance was limited: only two studies (21, 31) explicitly referenced the Transparent Reporting of a multivariable prediction model for Individual Prognosis or Diagnosis (TRIPOD) or TRIPOD+AI (updated guidance for prediction models incorporating AI/ML), which diminishes transparency and reproducibility (63, 64).

### 4.3 Implications for future research and clinical translation

Drawing on our findings, we propose several priorities. First, prospective, multicenter cohort studies with prespecified modelling plans and adequate sample sizes should be the norm; such designs mitigate selection bias and better capture population heterogeneity. Second, every novel model should undergo independent external validation in temporally and geographically distinct cohorts, and, where performance degrades, local recalibration or model updating should be performed and reported. Third, model reporting must adhere to established standards and bias assessments to ensure transparency and reproducibility.

Fourth, integrating multimodal data—serial clinical measures, pathology imaging, and emerging omics signatures—offers a plausible path to improved accuracy, but the added complexity must be justified by net clinical benefit and feasibility. In this context, machine-learning approaches may provide gains by capturing nonlinearities and interactions; however, they require careful attention to interpretability, rigorous external testing, and clear reporting of hyperparameters and training procedures to avoid opaque “black-box” solutions that clinicians cannot trust.

Finally, translational impact will depend not only on statistical performance but also on implementation studies that embed prediction tools within electronic health records, measure effects on clinician behavior, downstream management, patient outcomes, and cost-effectiveness. Only such implementation-effectiveness evidence can establish whether predictive tools actually reduce progression to ESRD or improve system efficiency.

At a minimum, future DKD-to-ESRD prediction studies should recruit clinically diagnosed DKD patients whenever feasible, prespecify a clinically relevant prediction horizon, and ensure that follow-up is long enough to capture that horizon. Because no guideline currently specifies the optimal follow-up duration for DKD-to-ESRD prediction, a pragmatic reference point is approximately 4 years, while longer follow-up may be needed for earlier-stage DKD cohorts (65). Candidate predictors should be routinely available in the intended clinical setting at the time the model is used. Studies should either prespecify a single ESRD definition or, when multiple ESRD definitions are clinically important, develop and report separate models for each definition rather than combining them into one endpoint. They should report both discrimination and calibration (including calibration plots, calibration intercept, and calibration slope), undergo independent external validation in temporally and geographically distinct cohorts, justify sample size using modern prediction-model methods rather than EPV alone, comply with TRIPOD or TRIPOD-AI, and evaluate clinical utility with decision-curve analysis whenever feasible.

### 4.4 Study limitations

This review has limitations. First, all 15 included studies were judged at high risk of bias using PROBAST−AI, predominantly due to retrospective designs, single−center sampling, lack of blinded predictor assessment, and inadequate calibration and external validation. Consequently, the certainty of evidence from the pooled AUCs is low, and our meta−analysis should be considered exploratory. We restricted our search to English- and Chinese-language publications, which may introduce language bias. Included studies were heterogeneous with respect to populations, outcome definitions, and modelling choices, complicating quantitative synthesis. Importantly, the included studies defined ESRD differently, including dialysis initiation, transplantation, eGFR thresholds, and composite endpoints that incorporated death, which are clinically and methodologically distinct outcomes and may have obscured meaningful differences in pooled performance. Our review focused on model development and validation metrics and did not assess the clinical effectiveness or cost-effectiveness of model-guided interventions. The predominance of included studies from Asian populations limits the generalizability of conclusions to other ethnic groups. Lastly, the limited number of independent validation studies constrained the statistical power of some subgroup and sensitivity analyses, thereby limiting the breadth of inferences that can be drawn.

## 5 Conclusion

Published DKD-to-ESRD models show promising discrimination in development and internal-validation cohorts, but they are not yet suitable for direct clinical implementation because of pervasive methodological limitations, extreme heterogeneity, and scarce independent external validation. Accordingly, these models should be viewed as exploratory starting points for future model development rather than as ready-to-use decision-support tools. Future prospective, multicenter studies with rigorous external validation and calibration, together with multimodal data integration, are urgently needed to improve robustness and transportability.

## Data Availability

The original contributions presented in the study are included in the article/**Supplementary Material**. Further inquiries can be directed to the corresponding author.
