# Supplementary material for: Prediction models for progression from diabetic kidney disease to end-stage renal disease: a systematic review and meta-analysis
Source: Front Endocrinol (Lausanne). 2026 May 20;17:1812362. doi: 10.3389/fendo.2026.1812362 (PMC13229689; doi:10.3389/fendo.2026.1812362)
Supplement: Supplementary file 1 [file DataSheet1.docx]

Supplementary Material

# Supplementary Tables and Figures

## Supplementary Tables

**Supplementary Table 1** The detailed search strategy for this study

| **Database** | **Search Criteria** |
| --- | --- |
| **Pubmed** | ("Diabetic Nephropathies"[MeSH Terms] OR "diabetic nephropath*"[Title/Abstract] OR "nephropathy diabetic"[Title/Abstract] OR "nephropathies diabetic"[Title/Abstract] OR "diabetic kidney disease"[Title/Abstract] OR "diabetic kidney diseases"[Title/Abstract] OR "kidney disease diabetic"[Title/Abstract] OR "kidney diseases diabetic"[Title/Abstract] OR "diabetic glomerulosclerosis"[Title/Abstract] OR "glomerulosclerosis diabetic"[Title/Abstract] OR "intracapillary glomerulosclerosis"[Title/Abstract] OR "Kimmelstiel-Wilson disease"[Title/Abstract] OR "Kimmelstiel Wilson disease"[Title/Abstract] OR "nodular glomerulosclerosis"[Title/Abstract] OR "glomerulosclerosis nodular"[Title/Abstract] OR "Kimmelstiel-Wilson syndrome"[Title/Abstract] OR "Kimmelstiel Wilson syndrome"[Title/Abstract])  AND  ("Kidney Failure, Chronic"[MeSH Terms] OR "renal failure chronic"[Title/Abstract] OR "chronic renal failure"[Title/Abstract] OR "chronic kidney failure"[Title/Abstract] OR "end stage kidney disease"[Title/Abstract] OR "kidney disease end stage"[Title/Abstract] OR "end stage renal disease"[Title/Abstract] OR "renal disease end stage"[Title/Abstract] OR "end stage renal failure"[Title/Abstract] OR "renal failure end stage"[Title/Abstract] OR "ESRD"[Title/Abstract])  AND  ("prediction model"[Title/Abstract] OR "risk prediction model"[Title/Abstract] OR "risk prediction"[Title/Abstract] OR "prognostic model"[Title/Abstract] OR "prediction tool"[Title/Abstract] OR "risk score"[Title/Abstract] OR "risk assessment"[Title/Abstract] OR "area under curve"[Title/Abstract] OR "AUC"[Title/Abstract] OR "roc curve"[Title/Abstract] OR "nomogram"[Title/Abstract]) |
| **Embase** | ('diabetic nephropathy'/exp OR 'nephropathies, diabetic':ab,ti OR 'nephropathy, diabetic':ab,ti OR 'diabetic nephropathy':ab,ti OR 'diabetic kidney disease':ab,ti OR 'diabetic kidney diseases':ab,ti OR 'kidney disease, diabetic':ab,ti OR 'kidney diseases, diabetic':ab,ti OR 'diabetic glomerulosclerosis':ab,ti OR 'glomerulosclerosis, diabetic':ab,ti)  AND  ('chronic kidney failure'/exp OR 'chronic kidney disease':ab,ti OR 'chronic kidney disorder':ab,ti OR 'chronic kidney insufficiency':ab,ti OR 'chronic nephropathy':ab,ti OR 'chronic renal disease':ab,ti OR 'chronic renal failure':ab,ti OR 'chronic renal insufficiency':ab,ti OR 'kidney chronic failure':ab,ti OR 'kidney disease, chronic':ab,ti OR 'kidney failure, chronic':ab,ti OR 'kidney function, chronic disease':ab,ti OR 'renal insufficiency, chronic':ab,ti OR 'chronic kidney failure':ab,ti)  AND  ('predictive model'/exp OR 'forecast model':ab,ti OR 'forecast modeling':ab,ti OR 'forecast modelling':ab,ti OR 'forecast simulation':ab,ti OR 'forecasting model':ab,ti OR 'prediction model':ab,ti OR 'predictive modeling':ab,ti OR 'predictive modelling':ab,ti OR 'predictive simulation':ab,ti OR 'predictive model':ab,ti) |
| **Cochrane Library** | ("Syndrome, Kimmelstiel-Wilson" OR "Glomerulosclerosis, Nodular" OR "Kimmelstiel-Wilson Syndrome" OR "Intracapillary Glomerulosclerosis" OR "Kimmelstiel-Wilson Disease" OR "Nodular Glomerulosclerosis" OR "Kimmelstiel Wilson Disease" OR "Diabetic Nephropathy" OR "Kidney Disease, Diabetic" OR "Kidney Diseases, Diabetic" OR "Nephropathy, Diabetic" OR "Diabetic Kidney Disease" OR "Diabetic Kidney Diseases" OR "Nephropathies, Diabetic" OR "Glomerulosclerosis, Diabetic" OR "Diabetic Glomerulosclerosis")  AND  ("End-Stage Renal Failure" OR "Disease, End-Stage Kidney" OR "End-Stage Renal Disease" OR "End Stage Renal Disease" OR "Renal Failure, End-Stage" OR "Renal Disease, End Stage" OR "End-Stage Kidney Disease" OR "Kidney Disease, End-Stage" OR "End Stage Kidney Disease" OR "Chronic Renal Failure" OR "Renal Failure, Chronic" OR "Renal Failure, End Stage" OR "Renal Disease, End-Stage" OR "Disease, End-Stage Renal" OR "ESRD" OR "Chronic Kidney Failure")  AND  ("prediction model" OR "risk prediction model" OR "risk prediction" OR "prognostic model" OR "prediction tool" OR "risk score" OR "risk assessment" OR "area under curve" OR "roc curve" OR "nomogram") |
| **Web of Science** | TS=(Diabetic Nephropathies OR "Nephropathies, Diabetic" OR "Nephropathy, Diabetic" OR "Diabetic Kidney Disease" OR "Diabetic Kidney Diseases" OR "Kidney Disease, Diabetic" OR "Kidney Diseases, Diabetic" OR "Diabetic Nephropathy" OR "Diabetic Glomerulosclerosis" OR "Glomerulosclerosis, Diabetic" OR "Intracapillary Glomerulosclerosis" OR "Kimmelstiel-Wilson Disease" OR "Kimmelstiel Wilson Disease" OR "Nodular Glomerulosclerosis" OR "Glomerulosclerosis, Nodular" OR "Kimmelstiel-Wilson Syndrome" OR "Kimmelstiel Wilson Syndrome" OR "Syndrome, Kimmelstiel-Wilson")  AND  TS=("Kidney Failure, Chronic" OR "Renal Failure, Chronic" OR "Chronic Renal Failure" OR "End-Stage Kidney Disease" OR "Disease, End-Stage Kidney" OR "End Stage Kidney Disease" OR "Kidney Disease, End-Stage" OR ESRD OR "End-Stage Renal Disease" OR "Disease, End-Stage Renal" OR "End Stage Renal Disease" OR "Renal Disease, End-Stage" OR "Renal Disease, End Stage" OR "Renal Failure, End-Stage" OR "End-Stage Renal Failure" OR "Renal Failure, End Stage" OR "Chronic Kidney Failure")  AND  TS=("prediction model" OR "risk prediction model" OR "risk prediction" OR "prognostic model" OR "prediction tool" OR "risk score" OR "risk assessment" OR "area under curve" OR AUC OR "roc curve" OR "nomogram") |

The database search was performed in four electronic databases: PubMed, Embase, Cochrane Library, and Web of Science, from inception to September 27, 2025. Since all databases require different syntaxes, specific search strategies were developed for each.

**Supplementary Table 2** PRISMA 2020 checklist

| **Section and Topic** | **Item #** | **Checklist item** | **Location where item is reported** |
| --- | --- | --- | --- |
| **TITLE** | | |  |
| Title | 1 | Identify the report as a systematic review. | Title page (article title) |
| **ABSTRACT** | | |  |
| Abstract | 2 | See the PRISMA 2020 for Abstracts checklist. | Abstract (structured abstract: Background, Methods, Results, Conclusions) |
| **INTRODUCTION** | | |  |
| Rationale | 3 | Describe the rationale for the review in the context of existing knowledge. | Introduction, first three paragraphs |
| Objectives | 4 | Provide an explicit statement of the objective(s) or question(s) the review addresses. | Introduction, final paragraph |
| **METHODS** | | |  |
| Eligibility criteria | 5 | Specify the inclusion and exclusion criteria for the review and how studies were grouped for the syntheses. | Materials and Methods > Inclusion criteria (Population, Index prediction model, Comparative models, Outcome, Timing, Setting) and Exclusion criteria |
| Information sources | 6 | Specify all databases, registers, websites, organisations, reference lists and other sources searched or consulted to identify studies. Specify the date when each source was last searched or consulted. | Materials and Methods > Data sources and search strategy; Supplementary Material |
| Search strategy | 7 | Present the full search strategies for all databases, registers and websites, including any filters and limits used. | Materials and Methods > Data sources and search strategy; Supplementary Material |
| Selection process | 8 | Specify the methods used to decide whether a study met the inclusion criteria of the review, including how many reviewers screened each record and each report retrieved, whether they worked independently, and if applicable, details of automation tools used in the process. | Materials and Methods > Study selection |
| Data collection process | 9 | Specify the methods used to collect data from reports, including how many reviewers collected data from each report, whether they worked independently, any processes for obtaining or confirming data from study investigators, and if applicable, details of automation tools used in the process. | Materials and Methods > Data extraction |
| Data items | 10a | List and define all outcomes for which data were sought. Specify whether all results that were compatible with each outcome domain in each study were sought (e.g. for all measures, time points, analyses), and if not, the methods used to decide which results to collect. | Materials and Methods > Data extraction |
|  | 10b | List and define all other variables for which data were sought (e.g. participant and intervention characteristics, funding sources). Describe any assumptions made about any missing or unclear information. | Materials and Methods > Data extraction |
| Study risk of bias assessment | 11 | Specify the methods used to assess risk of bias in the included studies, including details of the tool(s) used, how many reviewers assessed each study and whether they worked independently, and if applicable, details of automation tools used in the process. | Materials and Methods > Risk of bias and applicability assessment |
| Effect measures | 12 | Specify for each outcome the effect measure(s) (e.g. risk ratio, mean difference) used in the synthesis or presentation of results. | Materials and Methods > Data extraction and Statistical analysis |
| Synthesis methods | 13a | Describe the processes used to decide which studies were eligible for each synthesis (e.g. tabulating the study intervention characteristics and comparing against the planned groups for each synthesis (item #5)). | Materials and Methods > Statistical analysis |
|  | 13b | Describe any methods required to prepare the data for presentation or synthesis, such as handling of missing summary statistics, or data conversions. | Materials and Methods > Statistical analysis |
|  | 13c | Describe any methods used to tabulate or visually display results of individual studies and syntheses. | Materials and Methods > Statistical analysis; Figures 1–5; Tables 1–4; Supplementary Material |
|  | 13d | Describe any methods used to synthesize results and provide a rationale for the choice(s). If meta-analysis was performed, describe the model(s), method(s) to identify the presence and extent of statistical heterogeneity, and software package(s) used. | Materials and Methods > Statistical analysis |
|  | 13e | Describe any methods used to explore possible causes of heterogeneity among study results (e.g. subgroup analysis, meta-regression). | Materials and Methods > Statistical analysis |
|  | 13f | Describe any sensitivity analyses conducted to assess robustness of the synthesized results. | Materials and Methods > Statistical analysis |
| Reporting bias assessment | 14 | Describe any methods used to assess risk of bias due to missing results in a synthesis (arising from reporting biases). | Materials and Methods > Statistical analysis |
| Certainty assessment | 15 | Describe any methods used to assess certainty (or confidence) in the body of evidence for an outcome. | Not reported |
| **RESULTS** | | |  |
| Study selection | 16a | Describe the results of the search and selection process, from the number of records identified in the search to the number of studies included in the review, ideally using a flow diagram. | Results > Study selection; Figure 1 |
|  | 16b | Cite studies that might appear to meet the inclusion criteria, but which were excluded, and explain why they were excluded. | Results > Study selection |
| Study characteristics | 17 | Cite each included study and present its characteristics. | Results > Study characteristics; Tables 1–2 |
| Risk of bias in studies | 18 | Present assessments of risk of bias for each included study. | Results > Risk of bias and applicability assessment; Table 3; Figure 3 |
| Results of individual studies | 19 | For all outcomes, present, for each study: (a) summary statistics for each group (where appropriate) and (b) an effect estimate and its precision (e.g. confidence/credible interval), ideally using structured tables or plots. | Results > Model development approaches and performance; Table 2 |
| Results of syntheses | 20a | For each synthesis, briefly summarise the characteristics and risk of bias among contributing studies. | Results > Meta-analysis results; Results > Risk of bias and applicability assessment |
|  | 20b | Present results of all statistical syntheses conducted. If meta-analysis was done, present for each the summary estimate and its precision (e.g. confidence/credible interval) and measures of statistical heterogeneity. If comparing groups, describe the direction of the effect. | Results > Training-set models; Results > Validation-set models; Figures 4–5 |
|  | 20c | Present results of all investigations of possible causes of heterogeneity among study results. | Results > Subgroup analyses; Results > Sensitivity analyses |
|  | 20d | Present results of all sensitivity analyses conducted to assess the robustness of the synthesized results. | Results > Sensitivity analyses; Table 4 |
| Reporting biases | 21 | Present assessments of risk of bias due to missing results (arising from reporting biases) for each synthesis assessed. | Results > Training-set models (Egger’s test) |
| Certainty of evidence | 22 | Present assessments of certainty (or confidence) in the body of evidence for each outcome assessed. | Not reported |
| **DISCUSSION** | | |  |
| Discussion | 23a | Provide a general interpretation of the results in the context of other evidence. | Discussion > Principal predictors: from canonical renal indices to multidimensional integration; Model performance, validation, and translational barriers |
|  | 23b | Discuss any limitations of the evidence included in the review. | Discussion > Study limitations |
|  | 23c | Discuss any limitations of the review processes used. | Discussion > Study limitations |
|  | 23d | Discuss implications of the results for practice, policy, and future research. | Discussion > Implications for future research and clinical translation |
| **OTHER INFORMATION** | | |  |
| Registration and protocol | 24a | Provide registration information for the review, including register name and registration number, or state that the review was not registered. | Methods > Study design (PROSPERO CRD420251127778) |
|  | 24b | Indicate where the review protocol can be accessed, or state that a protocol was not prepared. | Methods > Study design (PROSPERO CRD420251127778) |
|  | 24c | Describe and explain any amendments to information provided at registration or in the protocol. | Not reported |
| Support | 25 | Describe sources of financial or non-financial support for the review, and the role of the funders or sponsors in the review. | Funding |
| Competing interests | 26 | Declare any competing interests of review authors. | Conflict of Interest |
| Availability of data, code and other materials | 27 | Report which of the following are publicly available and where they can be found: template data collection forms; data extracted from included studies; data used for all analyses; analytic code; any other materials used in the review. | Data Availability Statement; Supplementary Material (search strategies / PRISMA checklist) |

**Supplementary Table 3** PRISMA 2020 for Abstracts checklist

| **Section and Topic** | **Item #** | **Checklist item** | **Reported (Yes/No)** |
| --- | --- | --- | --- |
| **TITLE** | | |  |
| Title | 1 | Identify the report as a systematic review. | Yes |
| **BACKGROUND** | | |  |
| Objectives | 2 | Provide an explicit statement of the main objective(s) or question(s) the review addresses. | Yes |
| **METHODS** | | |  |
| Eligibility criteria | 3 | Specify the inclusion and exclusion criteria for the review. | Yes |
| Information sources | 4 | Specify the information sources (e.g. databases, registers) used to identify studies and the date when each was last searched. | Yes |
| Risk of bias | 5 | Specify the methods used to assess risk of bias in the included studies. | Yes |
| Synthesis of results | 6 | Specify the methods used to present and synthesise results. | Yes |
| **RESULTS** | | |  |
| Included studies | 7 | Give the total number of included studies and participants and summarise relevant characteristics of studies. | Yes |
| Synthesis of results | 8 | Present results for main outcomes, preferably indicating the number of included studies and participants for each. If meta-analysis was done, report the summary estimate and confidence/credible interval. If comparing groups, indicate the direction of the effect (i.e. which group is favoured). | Yes |
| **DISCUSSION** | | |  |
| Limitations of evidence | 9 | Provide a brief summary of the limitations of the evidence included in the review (e.g. study risk of bias, inconsistency and imprecision). | Yes |
| Interpretation | 10 | Provide a general interpretation of the results and important implications. | Yes |
| **OTHER** | | |  |
| Funding | 11 | Specify the primary source of funding for the review. | Yes |
| Registration | 12 | Provide the register name and registration number. | Yes |

## Supplementary Figures

**Supplementary Figure 1.** Leave-one-out sensitivity analysis for the pooled AUC of training-set models.


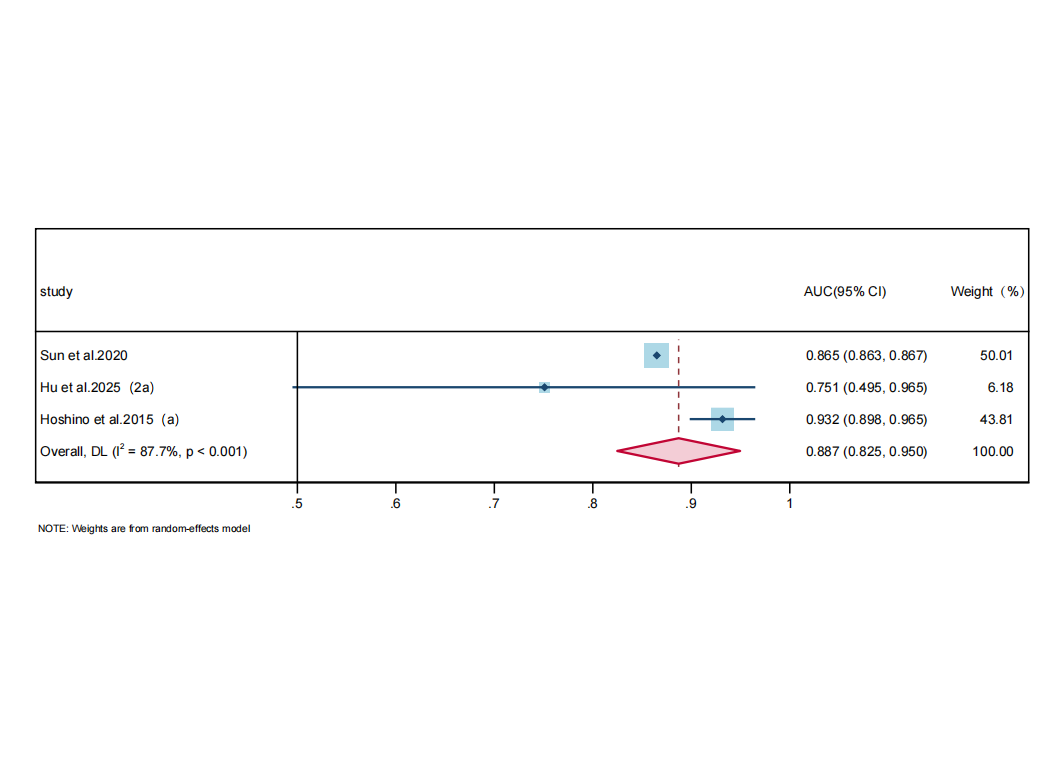


**Supplementary Figure 2.** Forest plot for subgroup of studies incorporating renal pathology predictors.


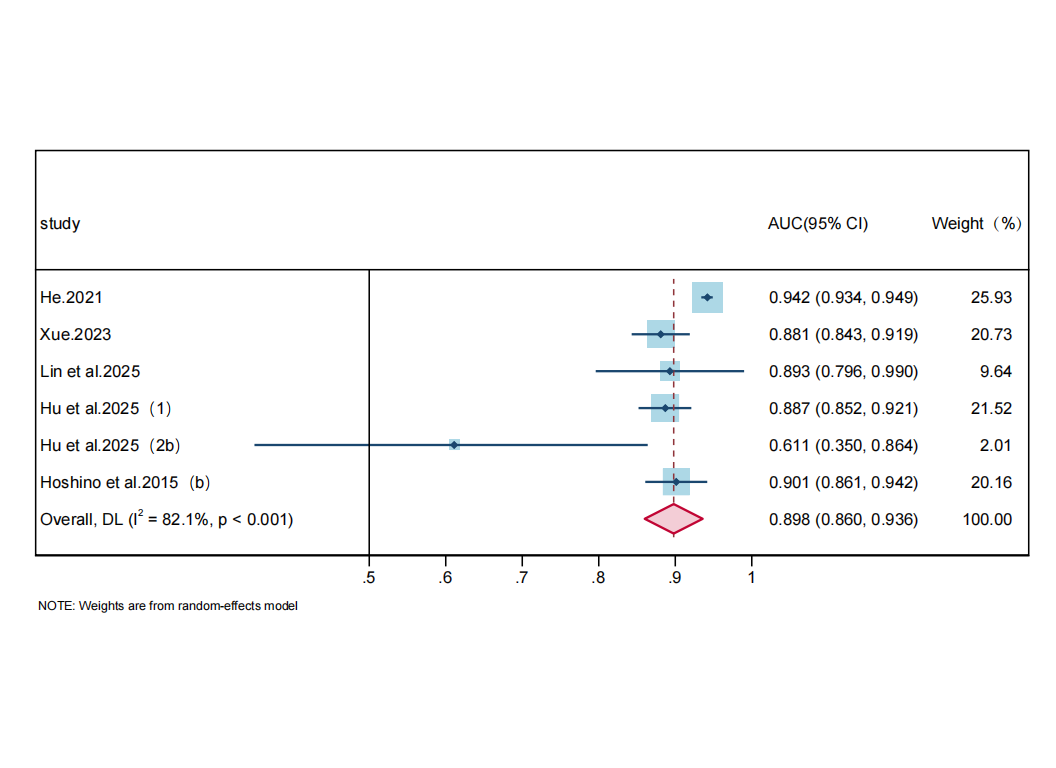


**Supplementary Figure 3.** Forest plot for subgroup of studies including only clinical predictors.


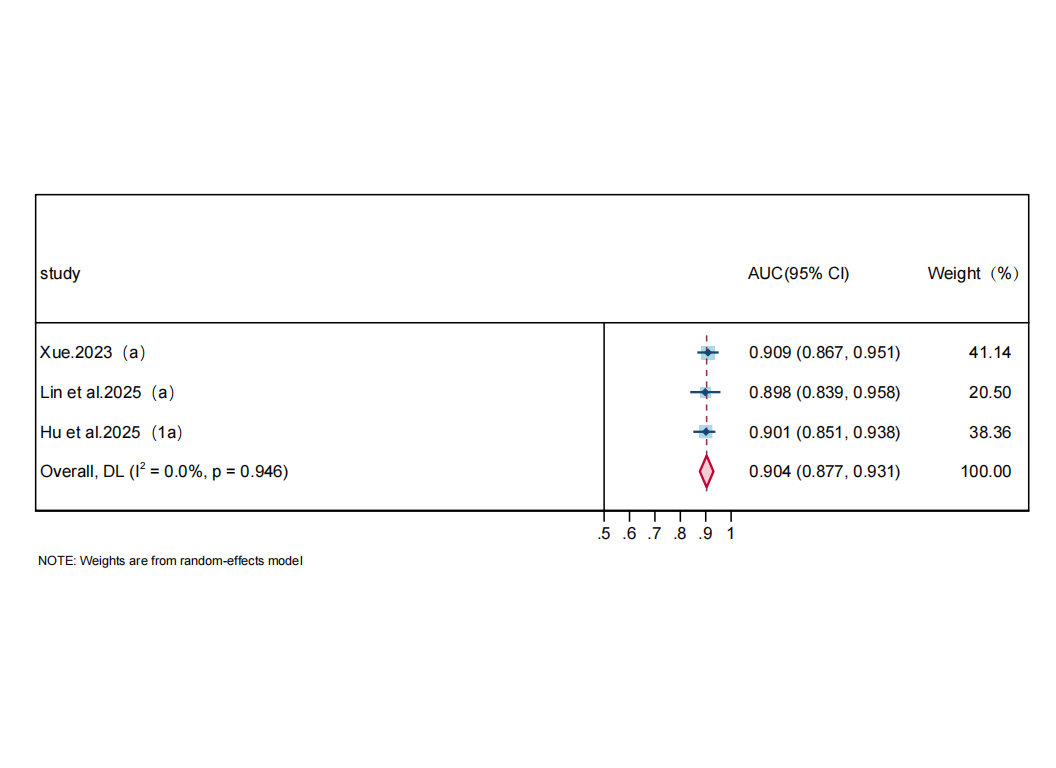


**Supplementary Figure 4.** Forest plot for subgroup of studies with 1‑year prediction window.


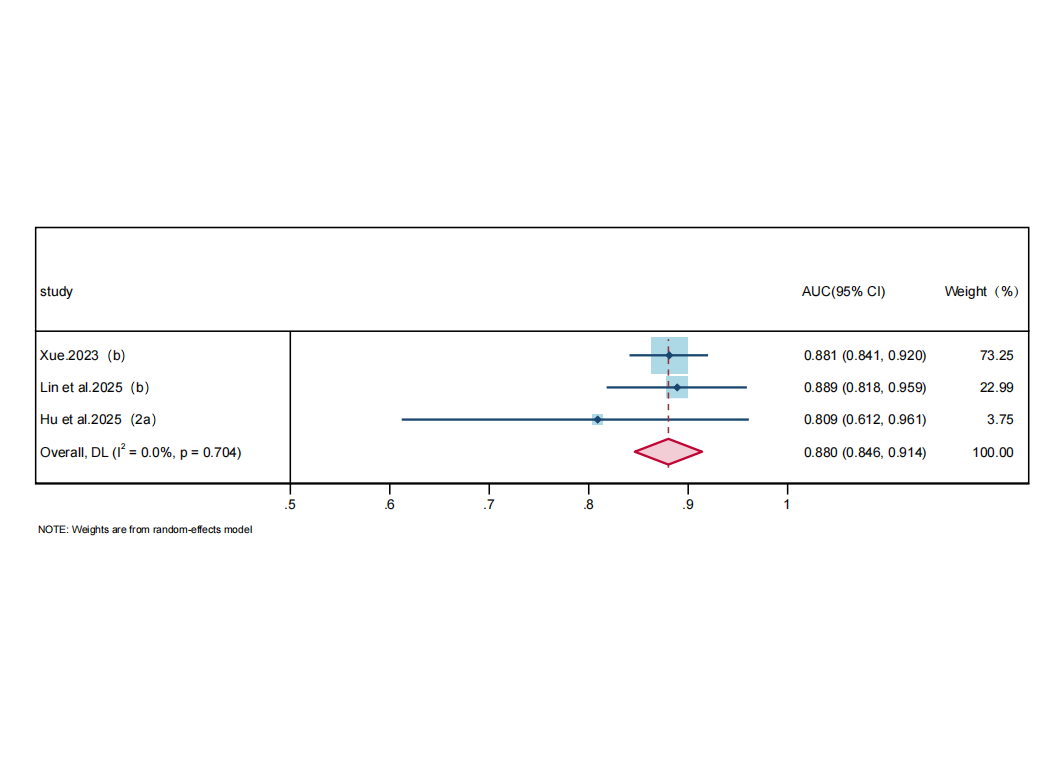


**Supplementary Figure 5.** Forest plot for subgroup of studies with 2‑year prediction window.


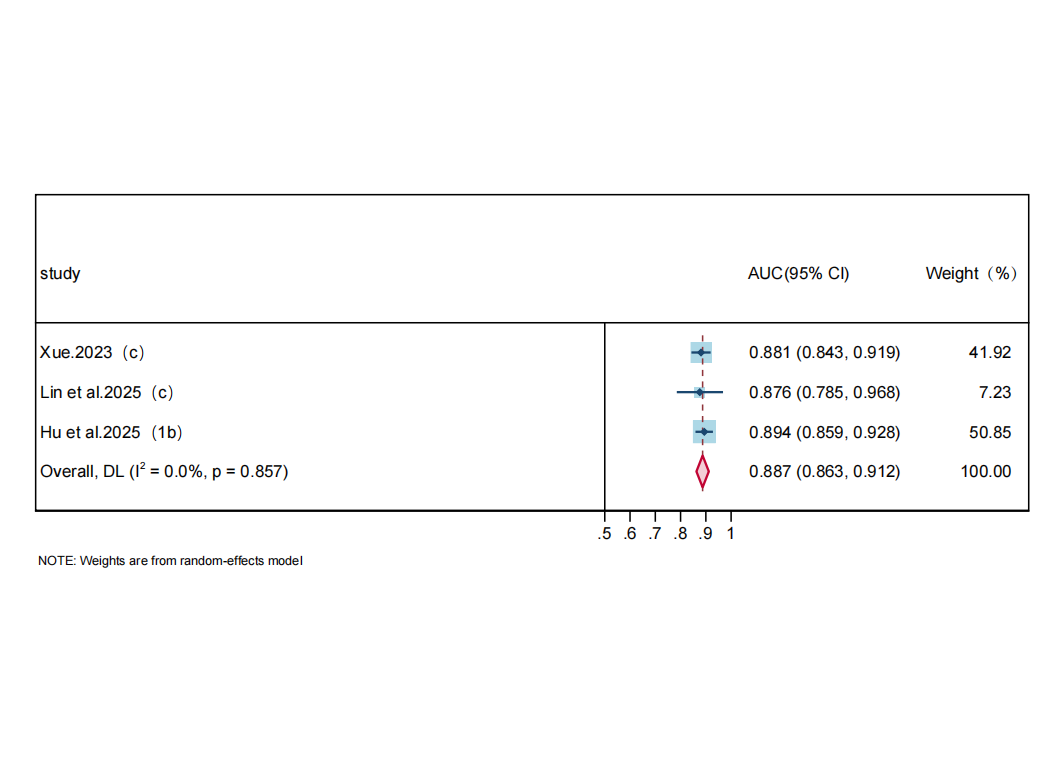


**Supplementary Figure 6.** Forest plot for subgroup of studies with 3‑year prediction window.


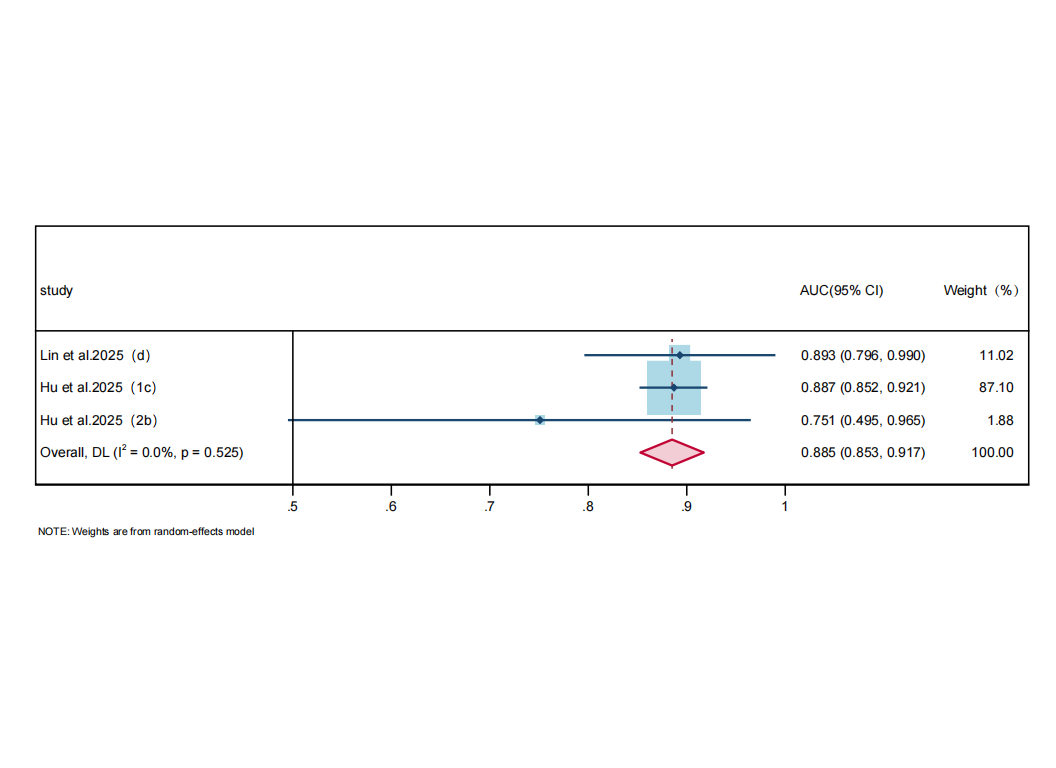


**Supplementary Figure 7.** Forest plot for subgroup of studies with 5‑year prediction window.


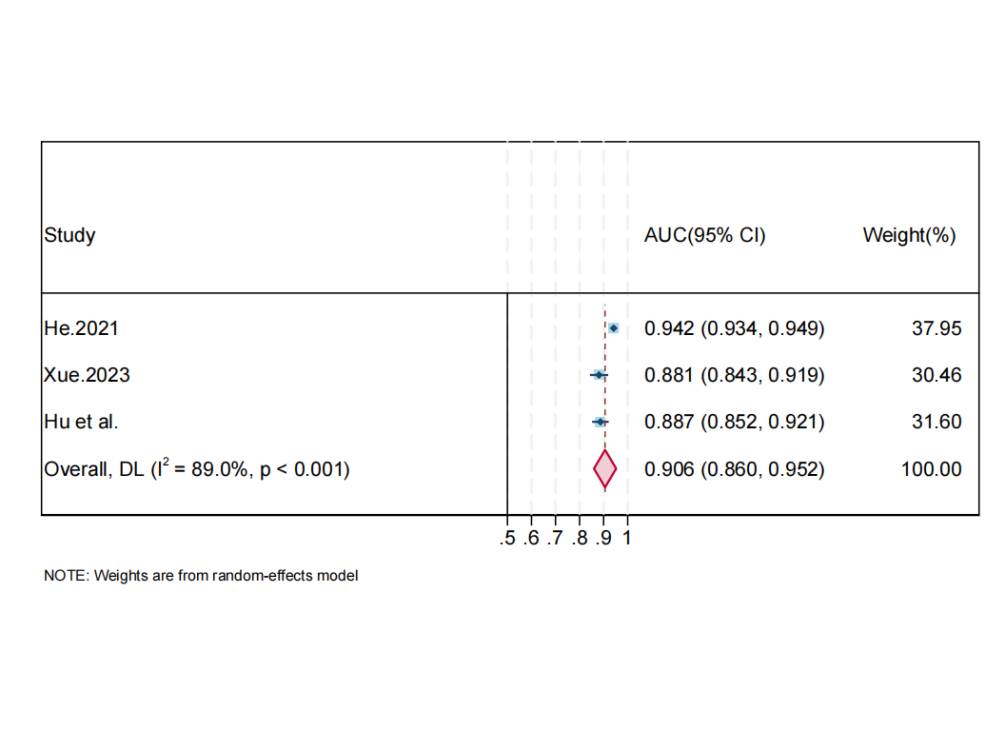


**Supplementary Figure 8.** Forest plot of the random-effects meta-analysis of 3 clinically diagnosed DKD training-set models.


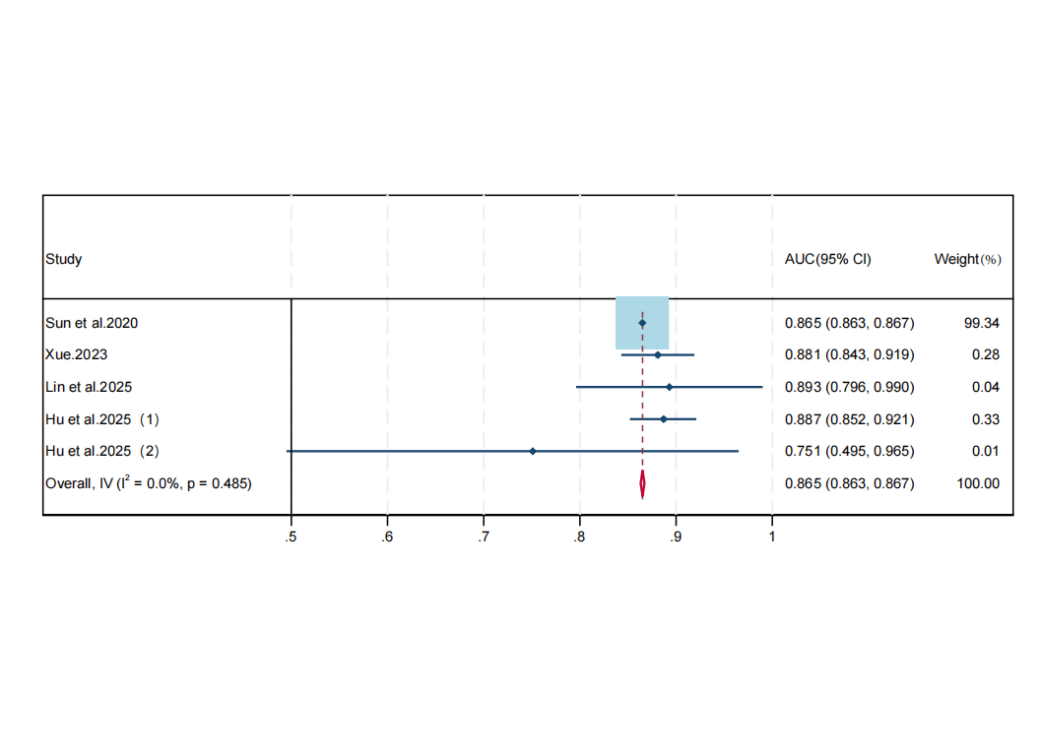


**Supplementary Figure 9.** Forest plot of the fixed‑effect meta‑analysis of 5 models using a composite ESRD definition.


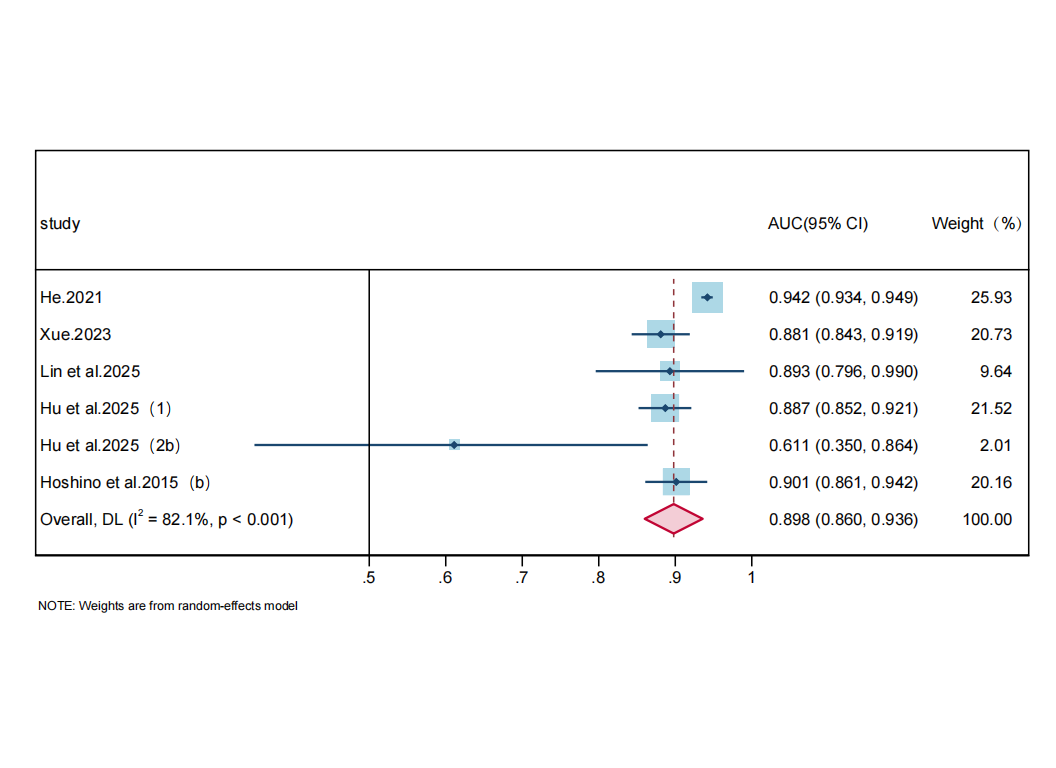


**Supplementary Figure 10.** Forest plot of the random-effects meta-analysis of 6 clinical-predictor-only training-set models.


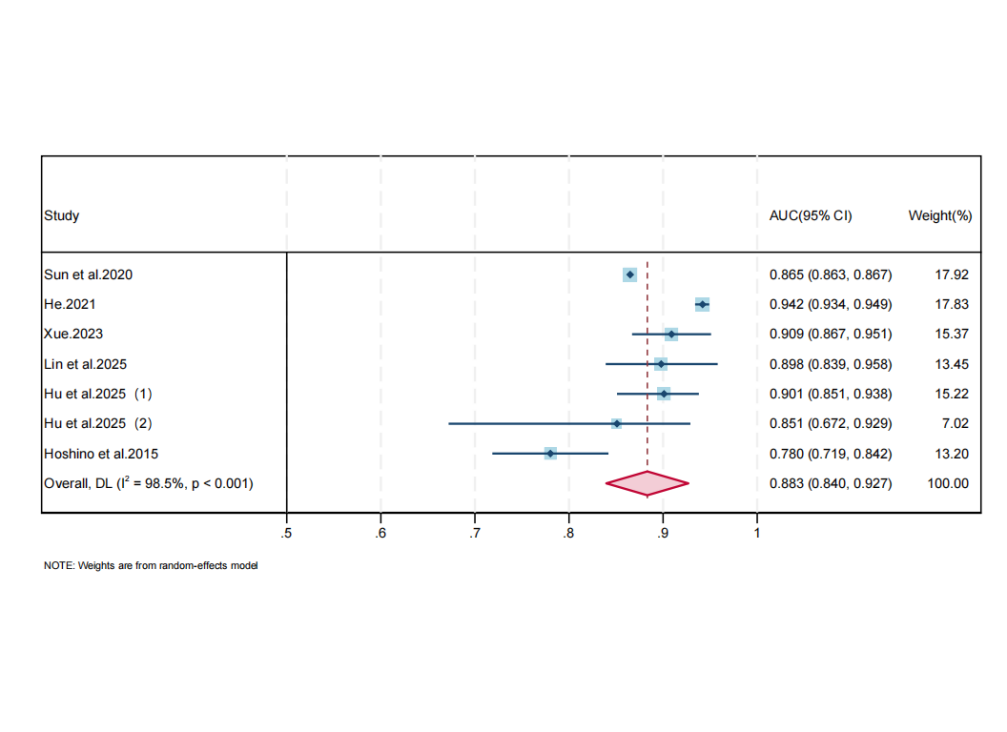


**Supplementary Figure 11.** Forest plot of the random-effects meta-analysis of 7 models selected using the alternative rule.

**
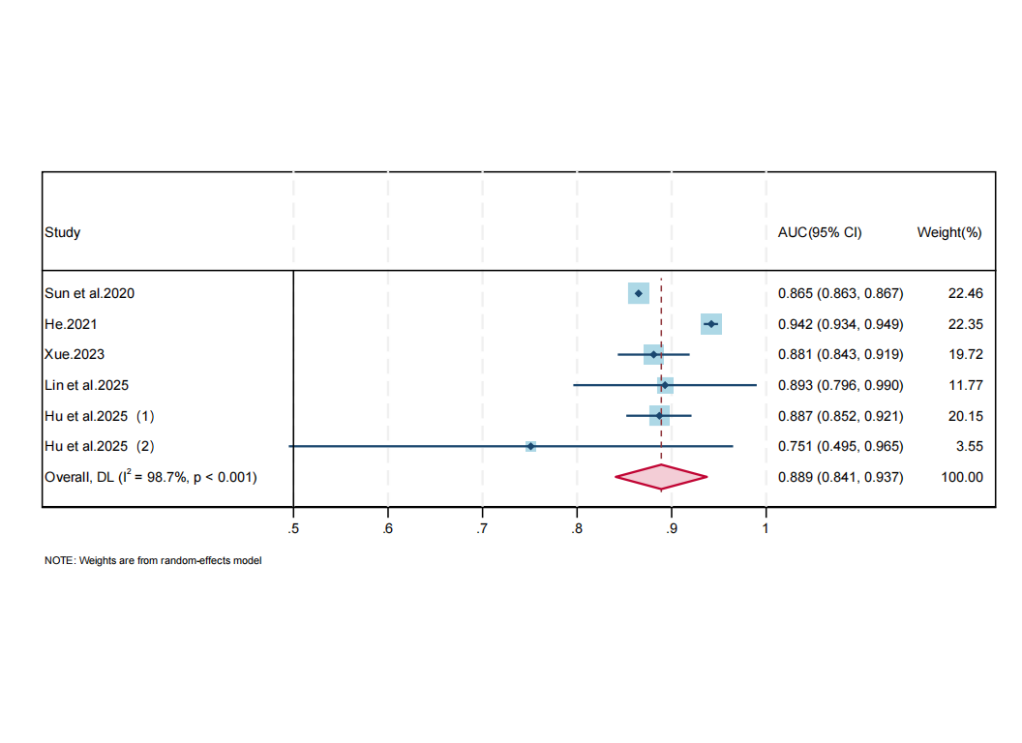
**

**Supplementary Figure 12.** Forest plot of the random-effects meta-analysis of 6 training-set models after excluding Hoshino et al. 2015, the study with the most concerning risk-of-bias profile.
